# Protective IgM-mediated immunity against *Vibrio anguillarum* in Atlantic cod with evolutionary losses of *mhc class II* and *cd4*

**DOI:** 10.3389/fimmu.2025.1579541

**Published:** 2025-07-02

**Authors:** Alexandra Jonsson, Adrián López-Porras, Simen Foyn Nørstebø, Naomi Croft Guslund, Henning Sørum, Shuo-Wang Qiao, Finn-Eirik Johansen

**Affiliations:** ^1^ Section for Physiology and Cell Biology, Department of Biosciences, The Faculty of Mathematics and Natural Sciences, University of Oslo, Oslo, Norway; ^2^ Department of Preclinical Sciences and Pathology, Faculty of Veterinary Medicine, Norwegian University of Life Sciences, Ås, Norway; ^3^ Department of Paraclinical Sciences, Faculty of Veterinary Medicine, Norwegian University of Life Sciences, Ås, Norway; ^4^ Institute of Clinical Medicine, Faculty of Medicine, University of Oslo, Oslo, Norway

**Keywords:** Atlantic cod, *Vibrio anguillarum*, IgM antibodies, antibody response, adaptive immunity, aquaculture

## Abstract

The unique adaptive immune system of the Atlantic cod (*Gadus morhua*), with genetic loss of the *mhc class II* and *cd4*, poses questions about the protective role of specific antibodies in this species. We investigate the IgM response against *Vibrio anguillarum*, a common pathogen in aquaculture. Juvenile Atlantic cod were bath-immunized with formalin-fixed *V. anguillarum* serotype O2a. Vaccinated cod were fully protected against lethal vibriosis when challenged by immersion with serotype O2a and partially protected against challenge with serotype O2b. Serum IgM from immunized cod reacted specifically with *V. anguillarum* O2a in ELISA, with some crossreactivity towards the O2b serotype. Bath-immunized cod were also protected against intracelomic challenge with *V. anguillarum* O2a, supporting the induction of systemic immunity by bath vaccination. Passive transfer experiments were conducted to evaluate the protective efficacy of IgM. Naive cod that received total serum or purified IgM from immunized donors were protected against lethal vibriosis; whereas, naive cod that received naive serum were not protected. Western blotting revealed that these protective antibodies recognized a proteinase K-sensitive antigen rather than lipopolysaccharides. These insights enhance our understanding of cod immunity and provide guidance for developing future vaccination strategies in aquaculture.

## 1 Introduction

Atlantic cod (*Gadus morhua*) is a vital species in the commercial fisheries of the North Atlantic, but overfishing has drastically reduced its population. As a result, aquaculture has become increasingly important to meet the demand for cod (1–3). Farming Atlantic cod presents considerable challenges, particularly due to their vulnerability to diseases. Among these, classical vibriosis caused by *Vibrio anguillarum* serogroups O2a and O2b remains a major bacterial threat in cod aquaculture (4–7).

Vaccination is the most cost-effective and sustainable strategy for managing fish diseases in farmed fish (8, 9), and an effective vaccine typically correlates with its ability to elicit robust antibody responses (10). However, studies show that Atlantic cod exhibit atypical antibody responses, which are often weak and do not correlate with survival following vaccination and challenge (11–17). This feature in cod may be linked to the loss of the MHC class II antigen presentation pathway, a hypothesis proposed by Pilström in 2005 (18). Genome sequencing by Star *et al.* in 2011 (19) later confirmed the genetic loss of the *mhc II* locus and the absence of a functional *cd4* gene. Further phylogenetic analysis suggests that these gene losses occurred in the ancestors of gadiform species approximately 100 million years ago (20). Despite these challenges, vaccination against vibriosis in Atlantic cod has proven successful and in addition, demonstrates the ability to induce cross-protection between serotypes O2, O2a, and O2b (17). Mikkelsen *et al.* (17) reported that vaccinated cod achieved full protection when challenged with a homologous strain of *V. anguillarum* and partial protection when infected with a heterologous strain. This suggests that the immune response in cod, although atypical, can effectively differentiate between serotypes and achieve a certain level of specificity (17).

The role of antibodies in specific immune protection in Atlantic cod remains unclear. In mammals, the CD4-MHC II axis is crucial for a normal antibody response, with affinity maturation and class switching both dependent on activation-induced deaminase encoded by the *aicda* gene (21). In Atlantic cod, this gene is speculated to be inactive, suggesting an inability to undergo affinity maturation (22). Cod generally exhibit high levels of natural serum IgM, regarded as constitutive, innate-like antibodies (23, 24) and this is the only secreted isotype identified in this species (24, 25). These polyreactive antibodies may provide broad-spectrum protection against various pathogens, as evidenced by cod serum IgM’s reactivity with some haptens (26, 27). Nevertheless, specific antibody responses are observed in cod when vaccinated with potent adjuvants, such as oil-based formulations; however, these responses are highly variable among individuals and primarily target lipopolysaccharide (LPS) antigens (16, 28–30). For example, Lund *et al.* (16) show that cod immunized with *A. salmonicida* bacterin or purified LPS in oil adjuvants raise specific antibodies against *A. salmonicida* LPS. Similarly, immunization with formalin-fixed *V. anguillarum* elicits sera reactive to lysate components, displaying the characteristic ladder pattern of LPS antigens (16, 29). In contrast, the failure of bath or dip immunization without potent adjuvants to induce specific antibodies raises questions about the role of antibodies in cod immune protection (11, 12, 17, 18). These findings emphasize the need to directly address the effect of antibodies in immune protection in Atlantic cod.

In this study, we investigated the protective role of specific IgM antibodies in Atlantic cod. We found that cod bath-vaccinated with *V. anguillarum* O2a serotype demonstrated full protection against homologous O2a and substantial cross-protection against heterologous serotype O2b. Notably, serum transfer experiments revealed that passive immunization with antibodies from vaccinated cod conferred protection to naive recipients, a novel finding in Atlantic cod research. This underscores the critical role of specific antibodies in protection, even in the absence of the MHC II and CD4 pathways. Furthermore, our study uncovered that the protective antibodies primarily targeted a protein antigen rather than the typically anticipated LPS. These findings provide further insights into the adaptive humoral immune response in Atlantic cod and offer practical information for developing targeted vaccine strategies to improve disease control in aquaculture.

## 2 Material and methods

### 2.1 Atlantic cod

Animal experiments were conducted per national and institutional guidelines and were approved by the Norwegian Food Safety Authority (FOTS ID21758, ID27682, ID30128). Atlantic cod larvae were purchased from NOFIMA Tromsø, Norway, and reared, vaccinated, and challenged at Norwegian Institute for Water Research (NIVA) facilities at Solbergstrand, Norway. Fish were maintained at water temperatures of 8-12°C in accordance with seasonal variation, a salinity of 34 PSU, light conditions of 12:12 hour light:dark, and fed with Skretting cod pellets. For each experiment, individuals from one or two single breeding families (one female and one male) were randomly allocated into different experimental and control groups. Prior to invasive procedures, fish were anesthetized for 5 min in a bath containing 50 mg/L tricaine methanesulfonate (FINQUEL^®^, MSD Animal Health). Blood samples were taken from the caudal vein using regular syringes.

### 2.2 Cultivation of bacterial strains

*V. anguillarum* O2a (ATCC19264 strain, from our strain library) and *V. anguillarum* O2b NVI 6078 (gift from Duncan Colquhoun, Veterinary Institute, Ås, Norway) were cultivated in Marine broth (Difco Marine Broth 2216) at 12°C with agitation (130 rpm). Bacterial inactivation was achieved by adding formalin to a final concentration of 0.5% and incubation at 4°C for 3 days with gentle shaking. Sterility was verified by plating out samples on blood agar plates [(Blood agar base no. 2; Oxoid, Cambridge, UK) with 5% ox blood added], with 2.5% NaCl and incubation at 18°C.

### 2.3 Immunization and challenge

Juvenile cod, aged 7 months and weighing 40–80 g, underwent vaccination via bath immersion with formalin-fixed *V. anguillarum* O2a. Water flow was halted for 30 minutes while inactivated bacterial suspensions were added to reach a final concentration of 5×10^6^ inactivated cells/mL under constant aeration. After 30 minutes, the water flow was restored to wash out the bacteria over several hours. A booster was similarly administered 4 weeks after the initial immunization. Infections were conducted 8–9 weeks after the booster, when fish weighed 70–200 g. For serum transfer experiments, larger cod (approx. 18 months at time of sacrifice, 300–700g) served as serum donors to obtain greater quantities of serum. These fish were vaccinated via bath immersion as described above, but received booster vaccinations at 4 weeks and 10 weeks after the initial priming, with serum collected 15 days post-final boost. Naive sera were collected at the same time from non-immunized siblings. Before conducting infection experiments, the virulence of *V. anguillarum* serotypes O2a and O2b was verified by intracelomic (i.c.) injection passage in Atlantic cod, followed by re-isolating from the head kidney to prepare challenge doses. A bath challenge was performed similarly to the immunization protocol with live bacteria for 1 hour, with halted water flow and constant aeration. Prior to the challenge experiments, optimal doses were determined through dose titration studies with naive cod. For the bath challenge, titrations were measured as CFU/mL for both serotypes (**Supplementary Figures 1A, B**), while the i.c. challenge with serotype O2a was measured as CFU/fish (**Supplementary Figure 1C**). The challenge dose used was 1×10^7^ CFU/mL for *V. anguillarum* O2a and 5×10^5^ CFU/mL for *V. anguillarum* O2b for the experiment assessing the specific protective efficacy of bath vaccination. For the i.c. challenge experiment, each fish received 0.3 mL of 1×10^5^ CFU/mL of *V. anguillarum* O2a in phosphate-buffered saline (PBS). For serum transfer experiments, 11-month-old juvenile cod (70–150 g) were injected i.c. with 0.3 ml serum derived from immunized donors (henceforth called immune serum), 0.3 ml of purified IgM from immune serum, or 0.4 ml serum from untreated donors (referred to as naive serum). The higher volume of naive serum was used to ensure the same quantity of IgM was transferred, as this serum pool had a slightly lower IgM concentration. A small sample (0.1%) of the amount injected per fish was analyzed by gel electrophoresis and Coomassie blue staining (**Supplementary Figure 2**). A bath challenge was performed 24 hours after serum transfer. For later experiments (serum transfer and control for i.c. challenge), bath challenge was conducted with a 5-fold higher bacterial concentration (5 × 10^7^ CFU/mL of *V. anguillarum* O2a) than the original bath challenge to ensure > 50% mortality.

### 2.4 Serum collection and purification of IgM

Atlantic cod were euthanized by a quick blow to the head and blood was immediately sampled from the caudal vein using a syringe. The blood was incubated for 2–4 hours at 8°C–12°C, centrifuged, and the serum was collected. Serum pools were prepared from 10 naive cod and from 33 bath-immunized cod, with each donor contributing an equal volume of serum. IgM was purified from a portion of the immune pool by two chromatography steps. First, crude immune serum was passed over a CM Affi-gel blue column (BioRad), as per Magnadóttir *et al.* (31). IgM-containing flow-through was collected, and IgM was precipitated by the addition of 0.313 g ammonium sulfate per ml. The precipitate was dissolved in 20 mM sodium phosphate, pH 7.5, and ammonium sulfate was added slowly to a final concentration of 1.0 M (0.132 g/ml). The solution was passed over a 5 ml HiTrap™ IgM column (Cytiva) with a syringe in batches to not exceed the binding capacity of the column, washed with 1 M ammonium sulfate, and eluted with 20 mM sodium phosphate, pH 7.5. Final eluate from several batches was pooled and precipitated as above, dissolved in PBS and dialyzed extensively against PBS in Slide-A-Lyzer™ Dialysis Cassettes, 10K MWCO (ThermoFisher). The volume was adjusted so that the purified IgM concentration was 2.5 mg/mL, equal to that of the crude immune serum pool. Purified IgM and crude sera were filter-sterilized using 0.45 *µ*m syringe filters before injection in the serum transfer experiment.

### 2.5 Enzyme-linked immunosorbent assay

To capture cod IgM from serum, mouse anti-cod IgM monoclonal antibodies (mAb) and chicken polyclonal antibodies to cod IgM were produced, as described elsewhere (Qiao et al., submitted manuscript). For total IgM ELISA, total chicken IgY anti-cod IgM (5 µg/ml), diluted in 50 mM carbonate buffer (pH 9.6), was used to coat Nunc MaxiSorp 96-well plates overnight (O/N) at 4°C. The remaining surface area was blocked with 5% skim milk (S) in PBS for a minimum of 90 minutes before washing three times with PBS/0.05% Tween 20 (T). All subsequent incubations and washing steps were performed in ELISA buffer (PBSTS) and PBST, respectively. After coating, titrated cod serum diluted 1:20,000 was added to the wells and incubated O/N at 4°C. For the detection of cod IgM, an in-house anti-cod IgM hybridoma supernatant mix diluted 1:10 was used as the primary antibody, and horseradish peroxidase (HRP)-conjugated goat anti-mouse IgG (G-21040, ThermoFisher) diluted 1:2,000 was used as the secondary antibody. Both antibodies were incubated at room temperature (RT) for 1 hour. The plates were then incubated with HRP substrate (TMB, either Mabtech or Merck ES001) until a visible blue color appeared, followed by the addition of 1 M HCl to stop the reaction. The plates were read in a plate reader at 450 nm. For *V. anguillarum*-specific ELISA, the plates were pre-coated with 5*µ*g/mL poly-L-lysine in 50 mM carbonate buffer (pH 9.6) O/N at 4°C. Subsequently, the wells were coated with formalin-fixed *V. anguillarum* O2a or *V. anguillarum* O2b in PBS diluted to an OD600 nm of approximately 0.5, as described by Mikkelsen *et al.* (17). The plates were centrifuged and incubated at RT for 1 hour or O/N at 4°C. The plates were then blocked as described above, followed by the addition of cod serum samples diluted 1:30. Incubation, washing, and detection were performed as described previously.

### 2.6 Western blot analysis

For Western blotting, *V. anguillarum* O2a or *V. anguillarum* O2b bacterial pellets were resuspended in PBS and prepared in 1x LDS sample buffer (Bolt™) with 10 mM DTT, heated to 75°C for 10 min, and loaded onto Bolt™ 4% to 12%, Bis-Tris, 1.0 mm gel (ThermoFisher). A proteinase K-treated fraction of *V. anguillarum* O2a was made using sonication of bacteria resuspended in PBS, followed by centrifugation at 4,600 g for 15 min at 4°C. The supernatant was sterile filtered with a 0.2-*µ*m filter and treated with proteinase K (125 *µ*g/ml, ThermoFisher) in PBS with 0.25% SDS for 2 h at 50°C. The proteinase K-treated fraction was prepared as above before electrophoresis. After electrophoresis, proteins were transferred to PVDF membrane (Immobilon®-P, Merck) by semi-dry transfer with NuPAGE™ Transfer Buffer (ThermoFisher) in a Bio-Rad Trans-BlotR Turbo™ system according to the manufacturer’s instructions or stained with SimplyBlue™SafeStain (ThermoFisher). PVDF membranes were blocked with 10% skim milk in PBS. Antibody incubations were in ELISA buffer and washed (3 times for 10 min) in PBST. The rabbit serum against *V. anguillarum* O2a was a gift from Duncan Colquhoun (Veterinary Institute, Ås, Norway). The rabbit serum was pre-adsorbed with *V. anguillarum* serotype O2b and used at 1:1,000 for western blotting. The detection antibody was HRP-conjugated goat anti-rabbit (A6154, Merck), used at a 1:2,000 dilution. Pooled cod sera were used at a 1:100 dilution and bound IgM was detected with our mAb #19.3 against cod IgM at 3 *µ*g/ml. The detection antibody was HRP-conjugated goat anti-mouse, used at a 1:1,000 dilution. After the final wash, the bound antibody was revealed with ECL (Cytiva, RPN2109) using a light-sensitive camera (Bio-Rad ChemiDoc MP Imaging System).

### 2.7 Statistical analysis

Statistical analyses were conducted using GraphPad Prism version 10.0.0 (San Jose, CA). The anti-*V. anguillarum* response was analyzed using the Mann–Whitney test. Kaplan–Meier survival curves were generated, and the survival outcomes at 15 days post-challenge were evaluated using the log-rank (Mantel–Cox) test. Statistical significance is indicated in each figure where relevant.

## 3 Results

### 3.1 *V. anguillarum* O2a bath-vaccinated cod demonstrates partial cross-protection against *V. anguillarum* O2b challenge

**Figure 1:**
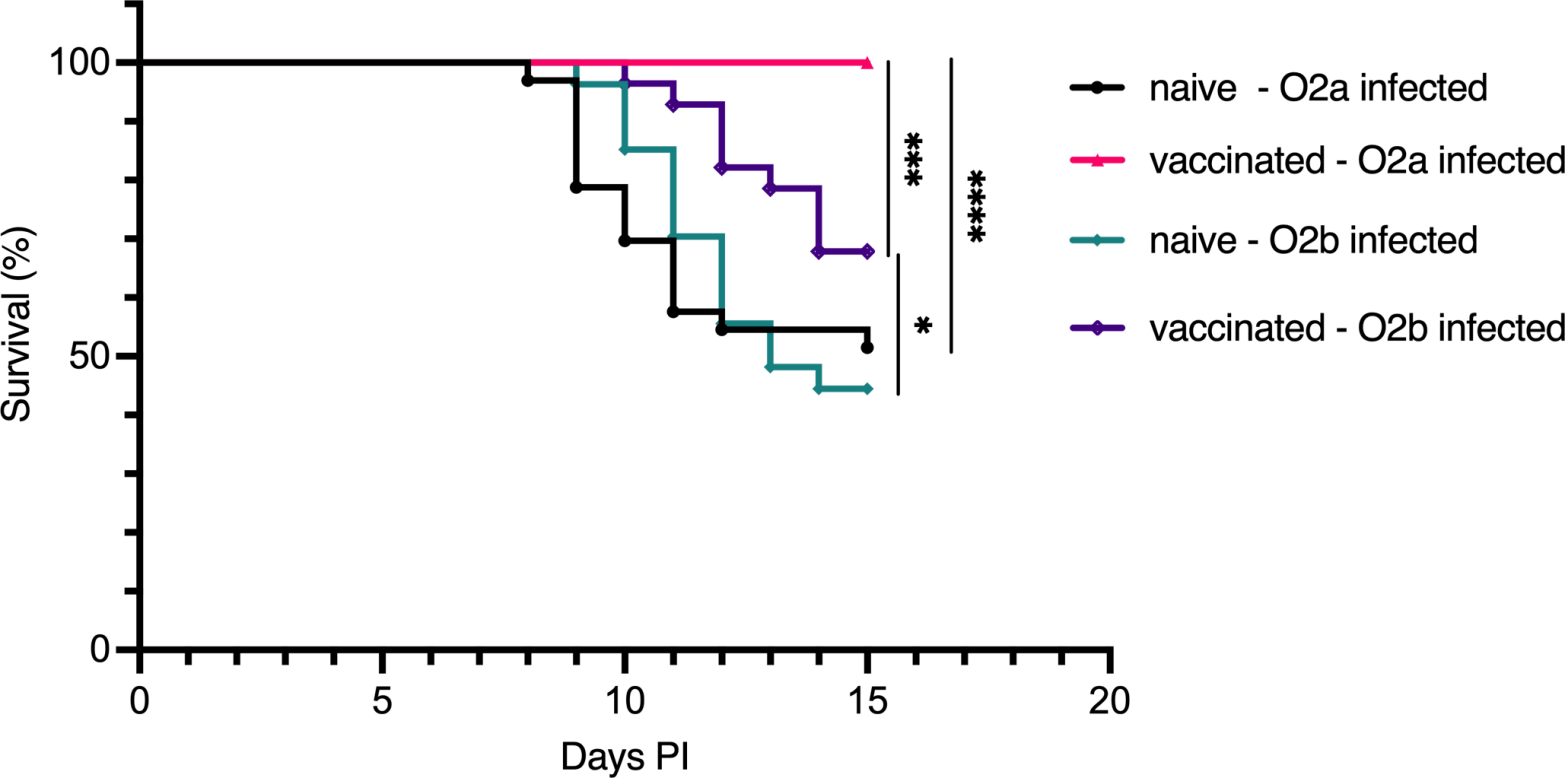
Survival of Atlantic cod following bath challenge with *V. anguillarum* O2a or *V. anguillarum* O2b. Survival curves for Atlantic cod bath-vaccinated with formalin-fixed *V. anguillarum* O2a and subsequently challenged with either O2a or O2b serotype. Four groups were assessed: naive cod infected with O2a (n=33), vaccinated cod infected with O2a (n=32), naive cod infected with O2b (n=27), and vaccinated cod infected with O2b (n=28). Statistical analysis of survival outcomes was conducted using the Mantel–Cox test, with significance indicated as follows: *p < 0.05, ***p < 0.0005, ****p < 0.0001.

Building on the findings of Mikkelsen *et al.* (17), we adapted their experimental protocol to assess the specific protective efficacy of bath vaccination in Atlantic cod. Juvenile cod were bath-vaccinated with formalin-fixed *V. anguillarum* serotype O2a, boosted 4 weeks later, and challenged via immersion 8–9 weeks after the boost with either *V. anguillarum* O2a or O2b. Survival outcomes were assessed in both the vaccinated and naive groups. Consistent with the findings of Mikkelsen *et al.*, our results showed that the vaccinated fish exhibited 100% survival when challenged with *V. anguillarum* O2a and 70% survival when challenged with *V. anguillarum* O2b. In contrast, naive fish demonstrated significantly lower survival rates of approximately 50% against both serotypes. These results confirmed the cross-protection afforded by bath vaccination against different serotypes of *V. anguillarum* (**Figure 1**).

### 3.2 Systemic protection following bath-vaccination with *V. anguillarum* O2a

**Figure 2:**
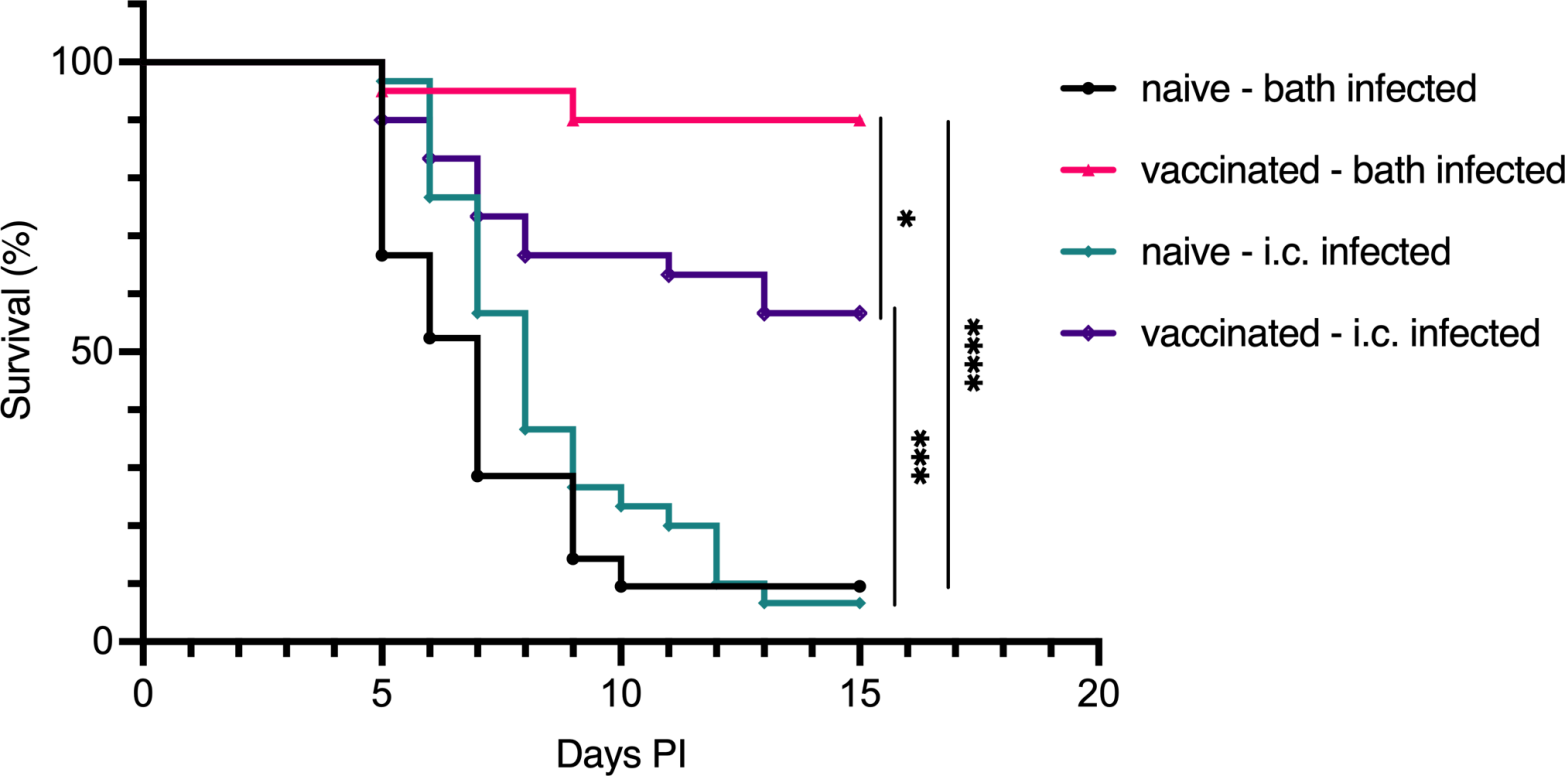
Survival of Atlantic cod after challenge with *V. anguillarum* O2a by i.c. injection. Survival curves for Atlantic cod, which were either bath-vaccinated with formalin-fixed *V. anguillarum* O2a or left naive, followed by challenge with the same serotype through two distinct routes: mucosal (bath) or systemic (i.c.) infection. Four groups were assessed: naive cod infected via bath (n=21), vaccinated cod infected via bath (n=20), naive cod infected via i.c. injection (n=30), and vaccinated cod infected via i.c. injection (n=30). Statistical analysis using the Mantel–Cox test yielded significance indicated by *p < 0.05, ***p < 0.0005, ****p < 0.0001.

The lethality of *V. anguillarum* infection in naive fish is closely related to the infective dose. To determine whether bath-vaccinated cod were protected primarily through an enhanced mucosal barrier function, resulting in reduced bacterial entry, or through a strengthened systemic immune response, we challenged vaccinated and naive Atlantic cod with *V. anguillarum* O2a via two routes: bath challenge (mucosal) and i.c. injection (systemic). Bath challenge or i.c. injection of naive cod resulted in a cumulative mortality of approximately 90% over 15 days. In contrast, bath-vaccinated cod exhibited significantly lower mortality rates, with 10% mortality following bath challenge and 43% following i.c. injection (**Figure 2**). This indicates that bath vaccination conferred substantial protection against both routes of infection, with more effective protection observed against bath challenge compared to i.c. injection.

### 3.3 Specific IgM antibody response in Atlantic Cod following bath immunization with formalin-fixed *V. anguillarum* O2a

**Figure 3:**
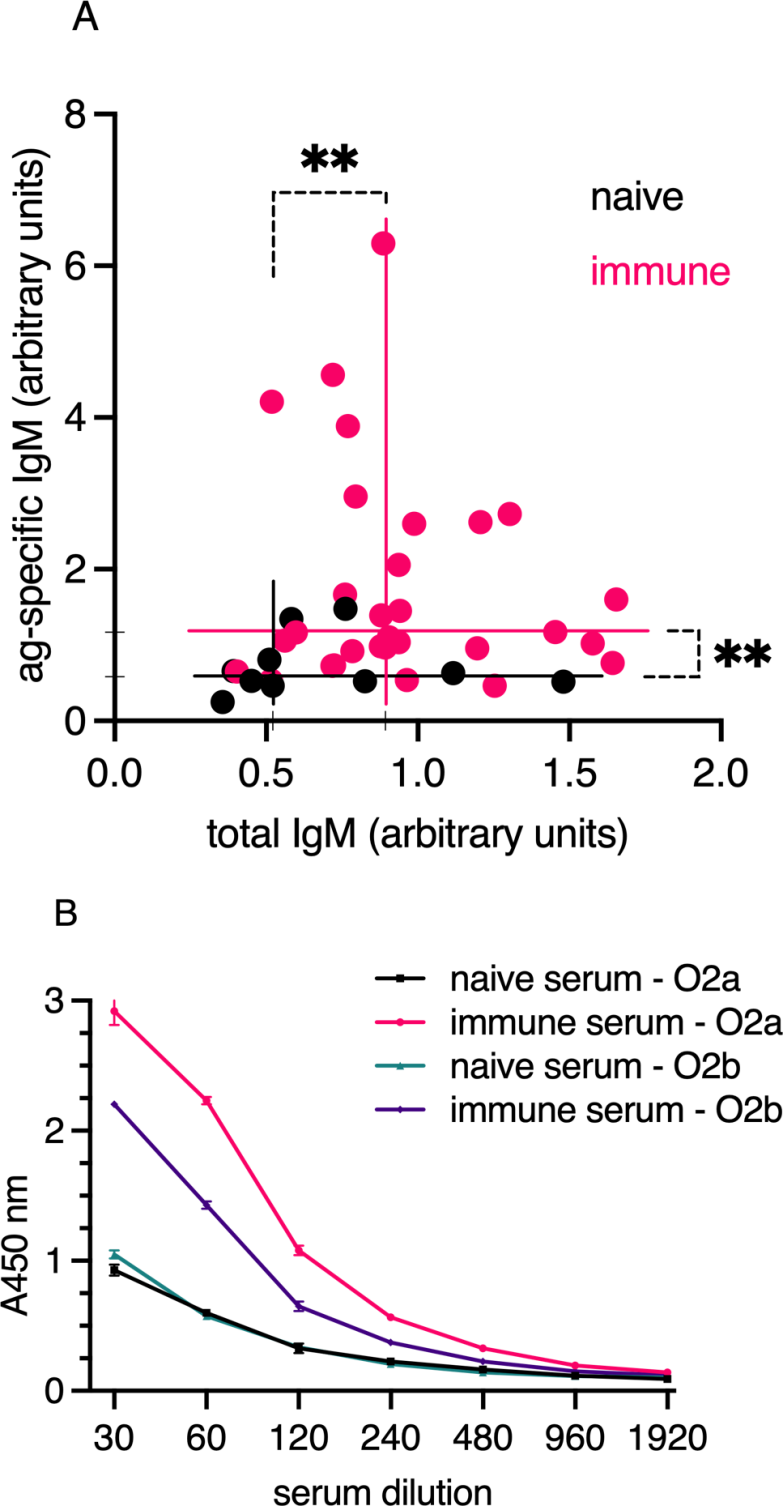
**(A)** Total IgM (x-axis) and antigen-specific IgM (y-axis) in serum from bath-immunized and naive cod were analyzed by ELISA. A pool of immune sera was used as a reference in the ELISA, equating to 1 arbitrary units/ml. Sample sizes were n=10 (naive); n=33 (vaccinated). The plot lines show median antigen-specific IgM levels of 0.58 for naive and 1.17 for immunized individuals on the y-axis, and total IgM levels of 0.52 for naive and 0.89 for immunized individuals on the x-axis. Statistical significance is indicated by ** for *p <* 0.005 (Mann–Whitney). **(B)** Serum IgM reactivity profiles against *V. anguillarum* serotypes O2a and O2b, measured by ELISA with pooled serum from bath-vaccinated *V. anguillarum* O2a or naive individuals. Black and green lines represent naive serum tested for reactivity to *V. anguillarum* O2a and O2b, respectively. Red and purple lines show binding to O2a and O2b, respectively, by immune serum. Serum IgM responses in Atlantic cod bath-vaccinated with *V. anguillarum* O2a.

To evaluate the ability of bath immunization to induce specific serum antibodies, we immunized a cohort of juvenile Atlantic cod with formalin-fixed *V. anguillarum* O2a. The immunization protocol included booster doses at 10 and 14 weeks following the initial immunization. Serum samples were collected 15 days after the final boost to measure *V. anguillarum* O2a-specific IgM and total IgM using both antigen-specific and total IgM ELISAs (**Figure 3**). The results demonstrated that bath immunization led to a significant increase in IgM reactivity against *V. anguillarum* O2a when compared to non-immunized, age-matched controls. Additionally, a notable elevation in total IgM levels was observed in the immunized group compared to the naive cohort. Approximately 8 of the 33 immunized individuals were identified as high responders for specific IgM, but these high responders did not exhibit elevated total IgM levels compared to the low responders in the vaccinated group (**Figure 3A**). Despite individual response variability, the overall group response, considering both specificity and total IgM concentration, was significantly higher than that of the naive group (n=10). This pattern was consistent with previous results from the prior year’s cohort, where antigen-specific responses were assessed in a vaccinated and boosted group that survived infection (**Supplementary Figure 4A**). Given the presence of specific IgM antibodies in immunized individuals, we assessed the cross-reactivity of pooled immune sera against *V. anguillarum* O2a and O2b. The pooled serum from vaccinated cod showed strong reactivity to *V. anguillarum* O2a and weaker cross-reactivity to O2b in ELISA assays (**Figure 3B**). In contrast, the naive serum exhibited lower reactivity compared to the immune sera and had similar background reactivity against both bacterial strains.

### 3.4 Serum antibodies in immunized Atlantic cod recognized proteinase K-sensitive antigens from *V. anguillarum* O2a

**Figure 4:**
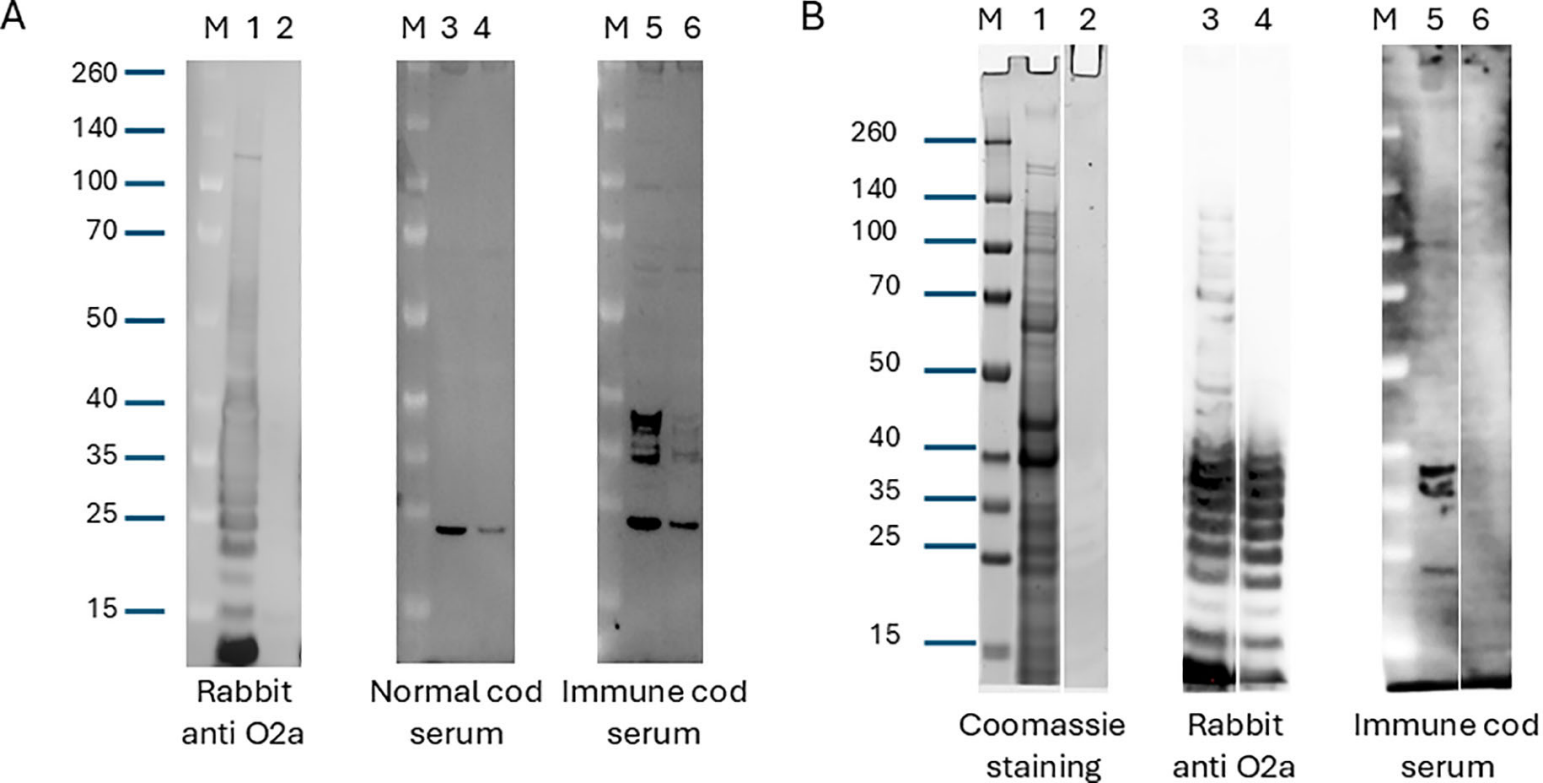
**(A)** Western blot analysis with rabbit serum against *V. anguillarum* O2a, naive cod serum, or cod serum from fish immunized with *V. anguillarum* O2a. Whole bacteria were grown in liquid culture, pelleted and solubilized in sample buffer, separated by gel electrophoresis, and transferred to a PVDF membrane. After transfer, the membrane was cut into different parts and probed with the indicated sera, followed by detection antibodies and HRP chemiluminescence. Lanes 1, 3, and 5 contain *V. anguillarum* O2a and lanes 2, 4, and 6 contain *V. anguillarum* O2b. The source of the primary antibodies is indicated below the blots. “M” denotes the molecular-weight size marker, and sizes (kDa) are indicated on the left. **(B)** Western blot analysis and Coomassie blue staining of three equal sets of samples made from pelleted whole *V. anguillarum* O2a (lanes 1, 3, and 5) or a sonicated and proteinase K-treated fraction of the same bacteria (lanes 2, 4, and 6). After electrophoresis, lanes 1 and 2 were stained directly with Coomassie blue while lanes 3–6 were transferred to a PVDF membrane. The PVDF membrane was cut after transfer and probed with the indicated sera. “M” denotes the molecular-weight size marker, and sizes (kDa) are indicated on the left. Western blot analysis of serum IgM reactivity in Atlantic cod immunized with *V. anguillarum* O2a.

To investigate the macromolecular composition of the antigens recognized by specific IgM in serum from Atlantic cod vaccinated without adjuvants, we used Western blot analysis. Solubilized cell pellets of *V. anguillarum* O2a and *V. anguillarum* O2b were probed with pooled sera from immunized and nonimmunized individuals. As a control, we probed an equivalent membrane with rabbit serum against *V. anguillarum* O2a, which had been pre-adsorbed with formalin-fixed *V. anguillarum* O2b. The rabbit serum reacted strongly with the antigens present in *V. anguillarum* O2a with very little cross-reactivity against *V. anguillarum* O2b (**Figure 4A**; lanes 1 and 2). The pooled cod serum from non-immunized fish reacted with a single band in *V. anguillarum* O2a and *V. anguillarum* O2b that had migrated to a position in the gel just below the 25 kD marker (**Figure 4A**; lanes 3 and 4). The pooled cod serum from immunized fish reacted with several antigens of *V. anguillarum* O2a that had migrated to a position in the gel equivalent to approximately 35–40 kD, and in addition reacted with the background band revealed by naive cod serum (**Figure 4A**; lane 5). Cross-reactivity against *V. anguillarum* O2b antigens in similar positions on the membrane was seen for both the specific and the non-specific (i.e., also present in naive serum) bands (**Figure 4A**; lane 6). This cross-reactivity may account for the partial cross-protection we observed when O2a-immunized fish were subjected to virulent *V. anguillarum* O2b (**Figure 1**). To further probe the molecular composition of the antigens that the immune cod serum showed specific reactivity towards, we ran side-by-side in the gel a sample from whole *V. anguillarum* O2a (as above) and a sample depleted in protein content and repeated the Western blotting (**Figure 4B**). Coomassie blue staining revealed that the proteinase K-digested sample showed significantly fewer and weaker bands than the whole bacterial lysate, demonstrating that a substantial amount of proteins were removed from this sample (**Figure 4B**; lanes 1 and 2). Protein digestion of *V. anguillarum* O2a did not reduce the intensity of staining with the rabbit anti-*V. anguillarum* O2a antiserum, suggesting that the antigen(s) detected with this serum were proteinase K-resistant (**Figure 4B**; lanes 3 and 4). Contrary to this, proteinase K digestion of *V. anguillarum* O2a significantly affected the antigens recognized by the pooled immune cod serum (**Figure 4B**; lanes 5 and 6).

### 3.5 Protection against lethal vibriosis by transferred IgM from bath-immunized cod

**Figure 5:**
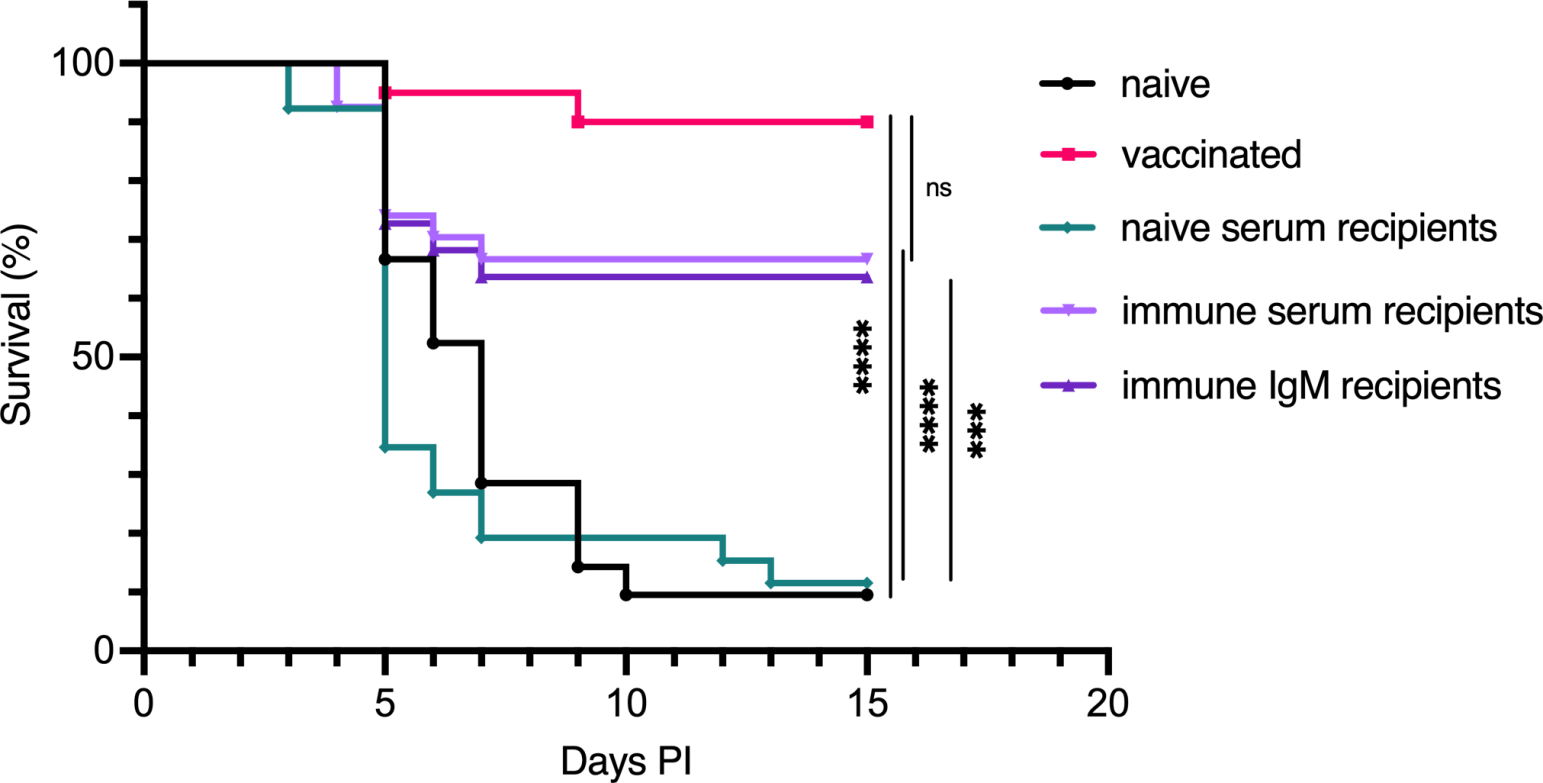
Protection against lethal vibriosis through the passive transfer of anti-O2a immune serum and purified IgM in Atlantic cod subsequently infected with *V. anguillarum* O2a. Survival curves for various treatment groups of Atlantic cod following challenge with *V. anguillarum* O2a: naive cod, bath-vaccinated, and naive cod that received either naive serum, immune serum, or purified immune IgM 24 hours before challenge. The sample sizes for each group are as follows: n=21 for naive, n=20 for vaccinated, n=26 for naive serum recipients, n=27 for immune serum recipients, and n=22 for immune IgM recipients. Survival in vaccinated fish was compared to the naive group, while the effects of immune serum or IgM were compared to the naive serum group using the Mantel–Cox test. Statistical significance is indicated as follows: *** for *p <* 0.0005, **** for *p <* 0.0001, with “ns” denoting not statistically significant. Control groups for naive and vaccinated bath-infected fish match those utilized in **Figure 2**.

Based on our findings that bath-vaccinated fish exhibited systemic protection and developed specific serum IgM antibodies targeting *V. anguillarum* proteinase K-sensitive antigens, we aimed to determine if these antibodies are crucial for protection against vibriosis. To test this, we conducted serum transfer experiments. In our pilot experiment, we used serum from cod that had been primed and boosted with *V. anguillarum* O2a, and had survived an infection, as the immune donor serum. Although the results did not reach statistical significance, there was a clear trend indicating that immune serum provided protection (**Supplementary Figure 3B**). This prompted us to repeat the experiment with a larger group of fish the following year. In this expanded experiment, our experimental design included comparing the protective effects of whole immune serum and purified IgM, providing clarity on the role of specific antibodies in mediating the protective response. Pooled serum from *V. anguillarum* O2a bath-immunized Atlantic cod, purified IgM from the same pool, or naive serum was injected i.c. into naive recipients. All the fish were injected with equivalent quantities of IgM, regardless of whether it came from naive serum, immune serum, or purified IgM. Furthermore, 24 hours after serum or IgM injection, the fish were bath challenged as before, and mortality was recorded for 15 days. We estimate that the concentration of donor antibodies in the serum of the recipients was only 0.8% ± 0.4% (mean ± standard deviation) compared to the concentration of those same antibodies in the donor (**Supplementary Figure 4**). This calculation is based on the redistribution of injected Eu-labelled IgM to the serum 24 hours after injection (27). The mortality of Atlantic cod injected with serum from naive donors was 90%, indistinguishable from that of naive cod (**Figure 5**). Conversely, immune serum and purified IgM from immune serum offered significant protection from lethal vibriosis, as both these groups showed only approximately 33% mortality. Fish actively immunized by two successive bath vaccinations demonstrated the best level of protection, with only 10% of fish in this group succumbing to infection.

## 4 Discussion

Understanding the role of antibodies in immune protection against bacterial infections is crucial for advancing vaccine efficacy. In Atlantic cod, the absence of the MHC class II-CD4 axis raises questions about their ability to mount specific and protective antibody responses. Although vaccination against vibriosis has demonstrated protection, it has not always correlated with measurable antibody levels, leading to debates about their significance. In this study, we demonstrated that specific IgM served as a major protective factor against vibriosis caused by *V. anguillarum* O2a, as shown through passive immunization experiments. Our findings revealed that these protective antibodies recognized a proteinase K-sensitive antigen rather than LPS, challenging previous assumptions from other studies.

Our study shows that bath vaccination with formalin-fixed *V. anguillarum* O2a provides significant protection to Atlantic cod against challenge with the homologous serotype and partial protection against the heterologous O2b serotype. This cross-protection suggests that the cod immune response can achieve a certain degree of specificity, allowing for differentiation between serotypes. These findings align with those reported by Mikkelsen *et al.* (17), who observed similar protective outcomes in their *in vivo* study, although they attributed the protection primarily to cell-mediated immunity due to a lack of measurable antibody titers (17). In contrast, our results show a significant increase in specific IgM levels, despite individual variability (**Figure 3A**), and demonstrate cross-reactivity with *V. anguillarum* O2b in both ELISA and Western blot, correlating with the survival outcomes observed. The overall response was statistically significant, although we noted overlapping reactivity between unimmunized and immunized fish. This overlap may explain why Mikkelsen’s study did not detect an antibody response to mucosal immunization. Differences in our experimental design, such as age and vaccination regime, may also explain this observation. In fact, humoral immunity has been shown to be affected by age and environmental parameters in Atlantic cod (32, 33).

Our results uniquely demonstrate specific antibody responses using immersion vaccination without adjuvants. This is noteworthy, given that previous studies have relied on potent adjuvants and injection methods to achieve similar results (16, 28, 30). While these studies successfully elicited specific antibodies, they also observed variability between individuals, with only a few high responders. The efficacy of adjuvants is well-documented in other teleost species (34), with different adjuvants producing varying titers and specificity, as seen in rainbow trout against *Aeromonas salmonicida* (35). Moreover, injections are regarded as the most effective vaccination route, providing long-lasting immunity through the depot effect, where antigens persist at the injection site (36). This effect has also been observed for *V. anguillarum* vaccinations in Atlantic halibut (37).

Immersion vaccination provided significant protection against i.c. injection of *V. anguillarum* O2a, but at a lower rate compared to the bath-challenged cohort. This suggests an extension of the vaccine-induced protection from mucosal sites to systemic compartments. In rainbow trout, mucosal vaccination has been shown to result in the presence of antibody-secreting cells in both the spleen and kidney, indicating possible systemic transport (38) as well as at mucosal sites, pointing to local immune processes (39, 40). In multiple teleost species, IgT and IgM are known to interact with the polymeric Ig receptor, facilitating their transport to mucosal surfaces (40–42). Our findings, together with other studies in teleost species, underscore the critical role of specific antibodies in providing effective immune protection across different compartments in Atlantic cod.

In earlier studies, the variability in antibody responses and the predominance of a few individuals with high antibody levels led to the notion that antibodies were not the main protective factor. While it might be tempting to attribute the observed antibody reactivity in the pooled serum against *V. anguillarum* O2a to this phenomenon, our findings suggest a broader protective effect. This conclusion is supported by our serum transfer experiments, which challenge previous assumptions regarding the role of antibodies in protection against *V. anguillarum*. The passive transfer of pooled immune serum and purified IgM from immunized donors conferred protection to naive recipients when they were subsequently challenged with *V. anguillarum* O2a (**Figure 5**). Since total IgM levels were higher in the immunized individuals, volumes of naive serum used as controls in passive transfer were adjusted to equalize the total IgM titers. Despite this, naive serum failed to protect against vibriosis. We estimate that the concentration of donor antibodies in recipient serum was approximately 100-fold less than the concentration of the same antibodies in the donors (**Supplementary Figure 4**), yet it was still sufficient for specific protection against vibriosis. It is a conundrum that the specific antibody titers in immunized fish far exceed what is typically necessary for protection against vibriosis, but detecting and quantifying these antibodies *in vitro* remains challenging. However, these findings are consistent with studies on rainbow trout, which demonstrate similar patterns of humoral and cell-mediated immune protection against *V. anguillarum* (43).

*V. anguillarum* exists in multiple serotypes, primarily distinguished by variations in the terminal O-linked oligosaccharides of their LPS (44, 45), However, genetic differences beyond LPS composition also contribute to serotype diversity (46). Previous studies have frequently documented antibody responses specific to LPS components. In our study, the serum obtained from *V. anguillarum* O2a-immunized cod reacted strongly with O2a serotype antigens and cross-reacted, albeit weakly, with similarly sized antigens from O2b bacteria. Western blot analysis revealed that protective antibodies interacted with distinct bands approximately 35–40 kD in size. These bands could initially be confused with LPS O-sugars based on size. However, proteinase K digestion of a cellular fraction from *V. anguillarum* O2a abolished the bands revealed by cod immune serum, but did not affect the LPS bands revealed by rabbit serum specific for *V. anguillarum* O2a. Thus, we suggest that the protective cod antibodies recognize a protein rather than LPS. Notably, a porin protein, classified as an outer membrane protein (OMP) of approximately 40 kDa, has been identified in at least 10 serovars of *V. anguillarum*, including serotype O2, and has been shown to elicit cross-reactive antibodies (47). This suggests that the protective antibodies from O2a-immunized cod target surface proteins rather than LPS, with IgM likely mediating cross-protection against O2b through reactivity with homologous proteins. Moreover, *V. anguillarum* O2a vaccination without adjuvants may exhibit specificity beyond LPS, potentially targeting other immunogenic antigens such as OMPs, flagellar proteins, and Type IV pili, all of which are of proteinaceous nature and have been implicated in cross-protective antibody responses (47–49). The absence of an antibody response against hapten-LPS, as noted in our recent study (27), supports the hypothesis that serotype-specific protection in Atlantic cod is mediated primarily by surface protein interactions, diverging from typical LPS-focused serotype-specific immunity observed in other species (50). A recent study by our group discovered that Atlantic cod utilizes a T cell-independent type II (TI-2) system for specific antibody induction (27). This mechanism offers insight into the paradox observed: while a soluble protein antigen failed to induce an antibody response (27), antibodies induced by formalin-fixed *V. anguillarum* recognized bacterial proteins. However, the proteinaceous antigen displayed by formalin-fixed bacteria could be an abundant bacterial surface protein, presenting as a repetitive hapten on the bacterial particle and thus fulfilling the requirements of a TI-2 antigen. Protein antigens are generally recognized as T cell-dependent antigens, whereas polysaccharides are T cell-independent antigens (51). However, OMPs have been shown to induce TI-2 antibody responses (52, 53).

The difficulty in detecting specific antibodies *in vitro* may stem from the nature of the antigens being TI-2, which are typically highly repetitive, rigid structures that act as complex conformational epitopes (54, 55). *In vitro* assays such as ELISA and Western blot often fail to capture these epitopes, as they rely on denatured or linearized antigens, which do not retain their native three-dimensional conformation (56). This explanation seems more likely than assuming that only a few fish successfully mounted an antibody response, while others relied on alternative immune mechanisms.

It is of interest to know the mechanism by which *in vivo* protection is mediated by vibrio-specific antibodies, but our efforts in this regard have thus far been unsuccessful. Enhanced complement-mediated bacterial lysis via the classical pathway or cell-mediated killing via interaction with Fc receptor-bearing immune cells are considered the main mechanisms of bacterial killing by antibodies. However, even in well-characterized model systems, determining the mechanisms of action of protective antibodies can be elusive. In a mouse model of *Coxiella burnetii* infection, *in vivo* protection against this intracellular bacterium was mediated by antibodies but did not depend on the complement or Fc receptors (57).

In summary, our findings reveal that specific IgM antibodies play a crucial role in protecting Atlantic cod against *V. anguillarum* O2a, targeting protein antigens rather than LPS. This finding challenges previous assumptions about antibody mechanisms in teleost fish lacking the MHC class II-CD4 axis. Future research should focus on characterizing the specific antigens involved, such as potential OMPs, and elucidating the effector mechanisms of these antibodies. These insights could significantly inform the development of targeted vaccines and enhance our understanding of teleost immune responses.

## Data Availability

The raw data supporting the conclusions of this article will be made available by the authors, without undue reservation.
