# Supplementary material for: Protective IgM-mediated immunity against Vibrio anguillarum in Atlantic cod with evolutionary losses of mhc class II and cd4
Source: Front Immunol. 2025 Jul 2;16:1579541. doi: 10.3389/fimmu.2025.1579541 (PMC12265950; doi:10.3389/fimmu.2025.1579541)
Supplement: Supplementary file 1 [file SupplementaryFile1.pdf]

Supplementary Material

1 SUPPLEMENTARY FIGURES

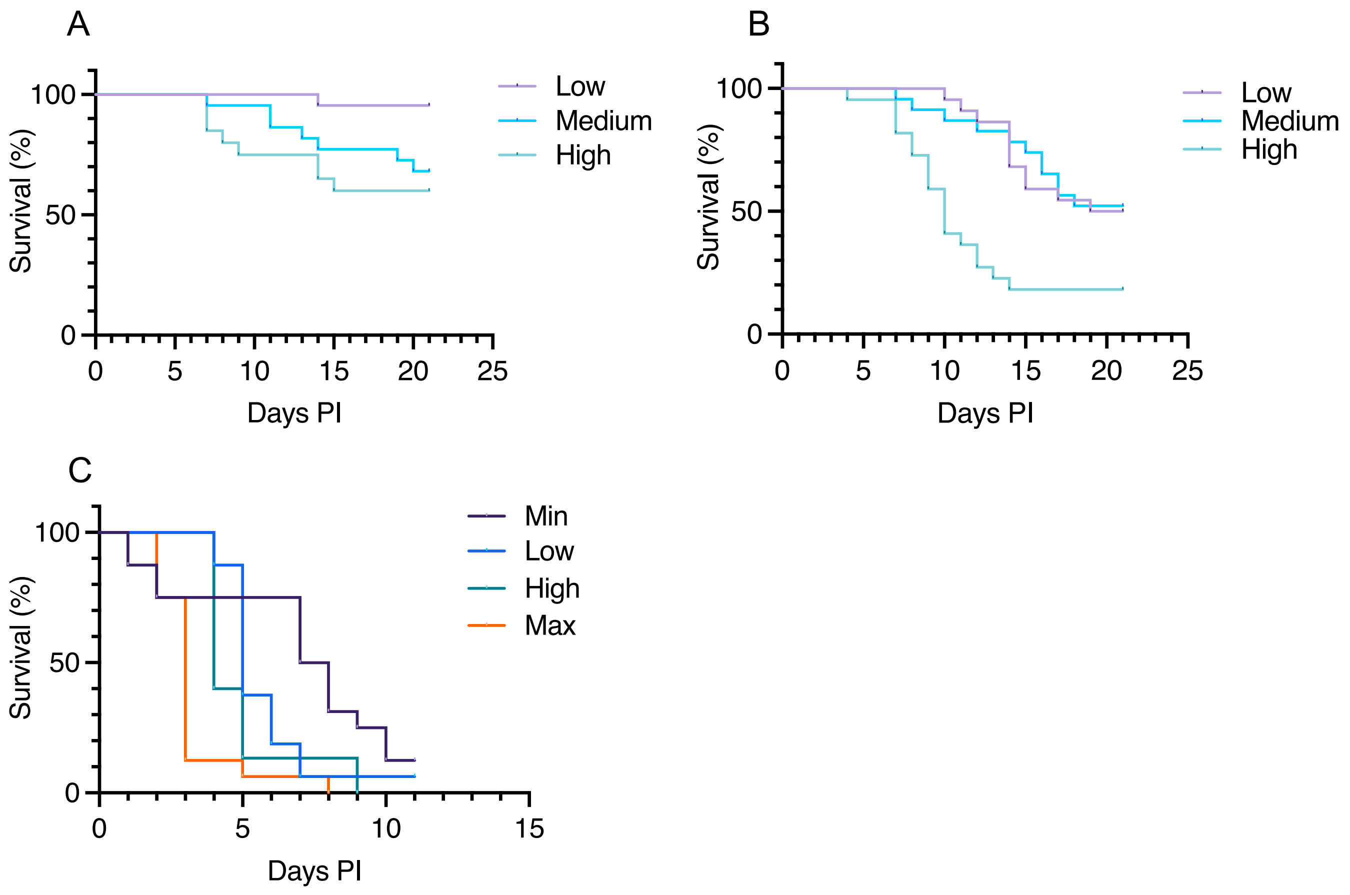

Figure S1. Survival outcomes in Atlantic cod following dose titration of *Vibrio anguillarum* infections. Survival curves for naive Atlantic cod subjected to bath infection with specified serotypes of *Vibrio anguillarum* using varying doses.(**(A)** Survival following bath infection with *V. anguillarum* O2a. Fish were divided into groups of n=22, with varying doses: Low (4.4E+04 CFU/ml), Medium (5.3E+05 CFU/ml), and High (5.3E+06 CFU/ml). **(B)** Survival following bath infection with *V. anguillaru*m O2b. Fish were divided into groups of n=22, with doses: Low (5.9E+04 CFU/ml), Medium (4.2E+05 CFU/ml), and High (5.0E+06 CFU/ml). **(C)**Survival following intracelomic infection with *V. anguillarum* O2a. Fish were divided into groups of n=16, with the following doses: Min (1.0E+05 CFU/fish), Low (5.0E+05 CFU/fish), High (2.5E+06 CFU/fish), and Max (1.25E+07 CFU/fish)..

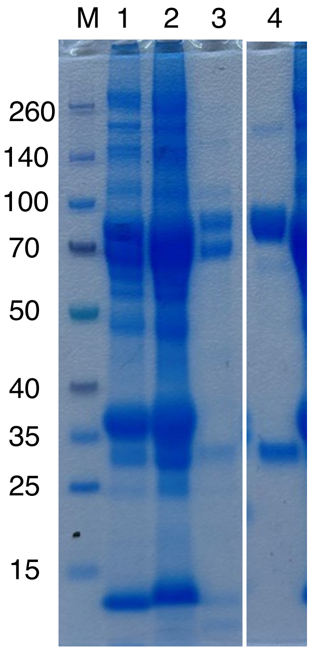

Figure S2. SDS PAGE analysis of serum samples and IgM prepared for injection in Atlantic cod. Samples were prepared in 1x LDS sample buffer (Bolt™) with 10 mM DTT, then heated to 75 ° for 10 min before being loaded onto a Bolt™ 4 to 12%, Bis-Tris, 1.0 mm gel (ThermoFisher). After electrophoresis, the gels were stained with SimplyBlue™ SafeStain (ThermoFisher). Lanes 1, 2, and 3 show the crude immune serum, crude naive serum, and partially purified IgM used in the serum and IgM transfer experiment, respectively. For each sample 0.1% of the amount injected per fish was run on the gel. Each recipient fish received 750 µg of IgM. For purified immune IgM and the immune serum pool, this amounted to 0.3 mL injected volume, while for naive serum pool, which had a slightly lower IgM concentration, this amounted to 0.4 mL injected volume. Lane 4 shows IgM purified by size exclusion chromatography for comparison. This is the IgM preparation, which was Europium labeled and used in injected experiments described Fig. S4. "M" denotes the molecular-weight size marker (kDa).

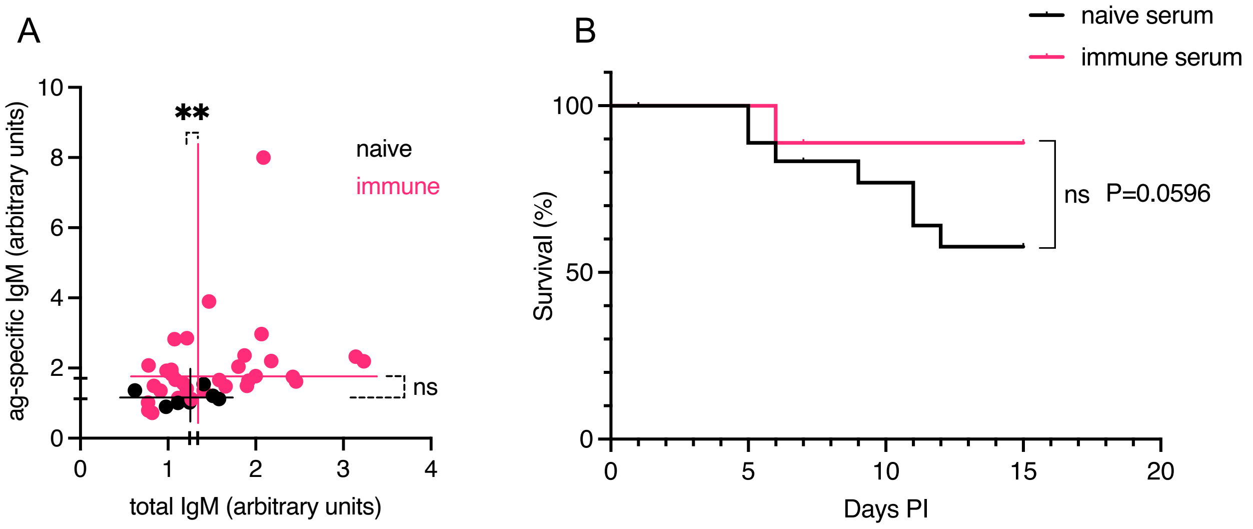

Figure S3. Serum IgM responses post-vaccination and pilot study of serum transfer in Atlantic cod following *V. anguillarum* O2a challenge(**(A)** ELISA showing ag-specific IgM levels (y-axis) versus total IgM levels (x-axis) in naive (n=7) and vaccinated (n=32) Atlantic cod. The immunized cod were bath-vaccinated with formalin-fixed *V. anguillarum* O2a. Specific IgM recognizing *V. anguillarum* O2a and total IgM levels were measured using a pool of immune sera as reference in the ELISA, equating to 1 arbitrary unit/ml. Statistical significance is denoted by ** for $p < 0.005$, with "ns" denoting not statistically significant based on the Mann-Whitney test.(B) This panel presents survival curves from a pilot serum transfer experiment, in which naive Atlantic cod recipients were injected with either naive or immune serum 24 hours before being challenged via bath infection with *V. anguillarum* O2a. This experiment was conducted one year before the main study, as shown in Figure 5. The immune serum used was harvested from vaccinated cod in a previous vaccination-challenge experiment, 15 days after infection. Serum was pooled, filter-sterilized, and 0.3 ml was injected intracelomically in each recipient. naive serum was obtained from cod of various ages without prior vaccination or infection. The results suggest a protective trend from immune serum, though it was not statistically significant (p=0.059), with both groups comprising n=16 fish. The serum used in this pilot study was obtained from the same individuals as those featured in panel A.

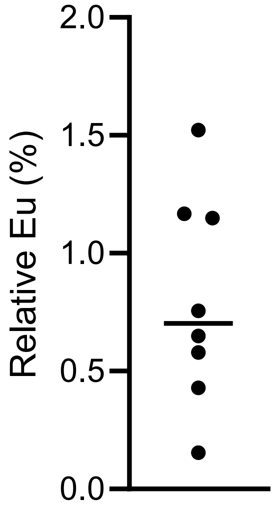

Figure S4. Estimation of serum transfer efficiency (specific activity of recipient compared with donor) To assess the level of specific IgM in recipient fish compared to donors, europium (Eu)-labeled purified cod IgM was used as a traceable surrogate as described inLópez-Porras et al. (2025). Briefly, a total of 1 mg of purified IgM was Eu-labeled following the manufacturer's protocol (PerkinElmer; DELFIA Sm-Labelling Kit 1244-303). Free Eu-chelate was removed using a G-50 spin column (Cytiva) and extensive dialysis against PBS. The labeled IgM was then adjusted to a volume of 3 ml with PBS, filter-sterilized, and prepared for injection. Eight Atlantic cod, each weighing approximately 70±30 g, received an intracelomic injection of 0.3 mL of Eu-labeled IgM. Blood samples were collected 24 hours post-injection, and the serum was isolated. The Eu-labeled IgM in both serum and injected material was quantified using a PerkinElmer Wallac 1420 Victor2 Microplate Reader, as per the DELFIA Sm-Labelling Kit instructions. The relative Eu activity/mL in each fish serum is expressed as a percentage of the Eu activity/mL of the injected material. The line on the graph represents the median level of 0.70%, with an interquartile range (I.C.R.) of 0.47%-1.16%

REFERENCES

López-Porras A, Jonsson A, Qiao SW, Johansen FE. Sustained t cell-independent type 2 antibody response in a naturally mhc ii-deficient teleost fish. *Developmental & Comparative Immunology* (2025) 105330.
